# Thermal sensation and comfort responses during repeated exposure to mild heat

**DOI:** 10.1186/s40101-025-00409-3

**Published:** 2025-11-27

**Authors:** Naoshi Kakitsuba, Kazuo Nagano

**Affiliations:** Department of Environmental Design, Kyoto Prefectural University, 1-5 Shimogamohangi-cho, Sakyo-ku, Kyoto, 606-8522 Japan

**Keywords:** Skin temperature, Thermal sensation response, Thermal comfort response, Sweating rate, Heart rate, Short-term heat acclimation

## Abstract

Since psychological and physiological responses to repeated exposure to mild heat has not been fully studied, the present study was designed to confirm overshooting responses in thermal sensation after repeated exposure to mild heat (i.e., the cooling period), the manner of change in the thermal sensation responses (TSRs) and the thermal comfort responses (TCRs) during the cooling period, and effect of short-term heat acclimation during repeated exposure to mild heat. In the summer, eight young adult male subjects (a mean age of 21.1 ± 1.4 years; a mean height of 173.1 ± 5.6 cm and a mean weight of 58.8 ± 7.5 kg) with clothing insulation (I_cl_, clo) of 0.3 clo first stayed in the control room at 26 °C for 15 min, then moved to the main testing room at 33 °C for 10 min (condition 1), 15 min (condition 2), or 20 min (condition 3), and finally returned to the control room for 15 min. The exposure was repeated five times. TSR and TCR were recorded in a 5-min interval from the beginning of the first exposure. The tympanic temperature (T_ty_), skin temperatures at the chest, forearm, front of the thigh, and front of the shin, and ECG and heart rate were continuously monitored. Local sweat rates at the same sites of skin temperature were monitored at the end of each exposure. Changes in T_ty_ and mean skin temperature ($\bar{\mathrm{T}}_{\mathrm{sk}}$) indicated no significant difference between conditions and no indication of short-term heat acclimation. Since the subjects voted nearly “cold” when $\bar{\mathrm{T}}_{\mathrm{sk}}$ remained high at the beginning of the cooling period, overshooting responses in thermal sensation were repeatedly observed in all conditions. The subjects voted “slightly cool” at the end of cooling period while $\bar{\mathrm{T}}_{\mathrm{sk}}$ kept decreasing during the cooling period. The thermally neutral $\bar{\mathrm{T}}_{\mathrm{sk}}$ was then estimated to be 0.3 °C—4.2 °C lower than $\bar{\mathrm{T}}_{\mathrm{sk}}$ observed prior to the first exposure. Thus, a residual effect on TSR during the cooling period was confirmed. Changes in the mean sweat rate, TSR and TCR showed significant differences between conditions but no indication of short-term heat acclimation. However, change in heart rate and ECG analysis implied the effect of short-term heat acclimation.

## Introduction

Heat stress and thermal comfort after a step change in air temperature were studied continuously in the past few decades. Horikoshi et al. [8] monitored physiological and psychological responses when young male subjects were first exposed to 23 °C and then to 15, 18, 23, 25, and 30 °C in the winter, and the participants demonstrated a difference in thermal sensation responses (TSRs) between 5 and 90 min after the step change in air temperature. Understanding the change in the TSR after a step change in air temperature, Kakitsuba and Inoue [11] reported overshooting responses of thermal sensation resulted from their experiment where the subjects voted “cool” at 27 °C soon after 40 °C exposure, and nearly “neutral” while $\bar{\mathrm{T}}_{\mathrm{sk}}$ decreased during step change in air temperature. The overshooting responses of thermal sensation can be observed during a step change from high to low air temperature. It is expected that people may vote “cool” or “slightly cool” soon after a step change*.* Recently, Chen et al. [3] also reported overshooting responses of thermal sensation due to the step change in air temperature. Takada et al. [18] then developed a model to predict the overshooting responses of thermal sensation in the non-steady state. However, since these experimental studies focused mainly on evaluating heat stress and the development of models of thermoregulation or thermal comfort, a single step change in air temperature was mainly adopted in the experimental designs.

Heat stress due to repeated exposure to heat or cold has also been studied but there are a few reports found in the literature. For example, Tokuda et al. [19] evaluated heat stress due to repeated exposure of older individuals to cold. Ohori et al. [16] reported large individual differences in heart rate variability (HRV) in response to an increase in outdoor air temperature when the subjects moved from indoors to outdoors in the summer and then demonstrated that heat stress may be correlated with HRV during a step change in air temperature.

Notley et al. [15] proved that repeated heat exposure in the summer enhanced heat loss in humans and reported that seasonal heat acclimatization enhanced evaporative heat loss in both young and older adults. Fujii et al. [5] reported short-term heat acclimation which may enhance whole-body heat loss by increasing evaporative heat loss after seven days of exercise. Bortolassi de Oliveira et al. [2] studied physiological responses to repeated heat exposure under equal workload conditions. Sixteen men were exposed continuously to the heat from a fire for 30 uninterrupted minutes and had intermittent exposure to the heat of the fire organized by two 15-min re-entries of exposure to the heat interspersed with 10 min of non-exposure. The results demonstrated that heart rate (HR) and other related variables in the re-exposure condition changed less than in the uninterrupted conditions. The results suggested that repeated exposure to heat with cooling period may reduce cardiac overload. Daanen et al. [4] reviewed heat acclimation which can be observed when the subjects exercised during repeated exposure to the heat for days or weeks. However, short-term acclimation during repeated expose to heat in a few hours has not been confirmed yet.

As mentioned earlier, an overshooting response of thermal sensation after a single hot exposure was reported by a few scientists but overshooting responses of the thermal sensation after repeated exposure to heat have not been fully confirmed. In the present study, considering the above backgrounds, the human experiment was designed to confirm overshooting responses in thermal sensation after repeated exposure to mild heat (i.e., the cooling period), the manner of change in the TSRs during the cooling period, and short-term heat acclimation during repeated exposure to mild heat.

## Methods

### Subjects

Repeated exposures to heat were conducted in the climate rooms at Meijo University from August to early September 2018. Eight healthy young Japanese male subjects with a mean age (± standard deviation, SD) of 21.1 ± 1.4 years participated in the experiment. A mean height and a mean weight were 173.1 ± 5.6 cm and 58.8 ± 7.5 kg, respectively. Since the sample size was limited mainly because of a prolonged period of a single exposure, power calculation was not conducted in the present study.

The subjects were required to wear a short-sleeved shirt and knee-length trousers. The clothing insulation of the ensembles was estimated to be 0.30 ± 0.13 clo from the equation proposed by Hanada [7]. To avoid effect of the subject’s circadian rhythm on the core temperature, the subjects were requested to follow a regular schedule for 1 week leading up to our experiment. Each subject routinely went to sleep before midnight and woke up at 07:00 to 07:30 each day.

Each subject provided written informed consent to participate in this study and was fully aware that they could withdraw from the study at any time without prejudice. The study protocol was approved by Meijo Institutional Ethics Review Board.

### Experimental protocol

**Fig. 1:**
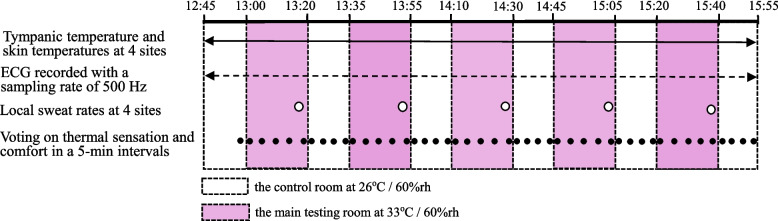
An example of the experimental protocol in condition 3. The experimental protocol in condition 3 is indicated as an example. The experiment started at 13:00 in all conditions. In this case, the subjects were exposed to 33 °C/60% RH in the main testing room and then returned to the control room at the end of 20 min exposure. Exposure to heat was repeated five times. The cooling period in the control room was set at 15 min in all conditions. Psychological and physiological responses were monitored continuously or periodically

The experiment started at 13:00. Air temperature (T_a_) and relative humidity (RH) in the control room were controlled at 26 °C/60% RH. The subjects moved to the main testing room to be exposed to 33 °C/60% as a single exposure and then returned to the control room at the end of each exposure. The cooling period in the control room was set at 15 min in all conditions. The exposure time to heat was set at 10 min (condition 1), 15 min (condition 2), and 20 min (condition 3). Exposure to heat was repeated five times in all conditions. As an example, the experimental protocol in condition 3 is indicated in Fig. 1.

### Measurements

Each subject was asked to respond to their perception of temperature and comfort. As shown in Table 1, a 9-point scale was used to assess thermal sensation, and a 4-point scale was used for thermal comfort as recommended by Gagge et al. [6]. Each subject reported their responses at 5-min intervals during each time period, including the end of and the beginning of each exposure, as shown in Fig. 1.

**Table 1:**
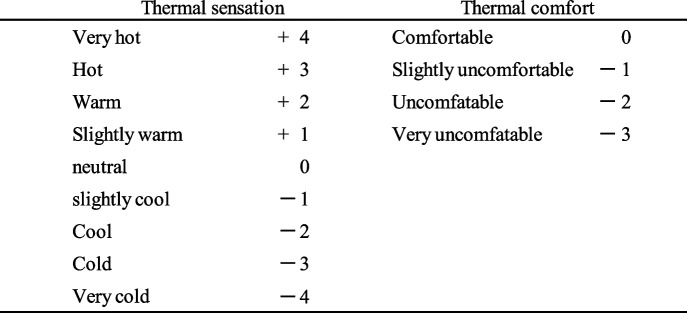
Categories for thermal sensation response (TSR) and thermal comfort response (TCR)

Tympanic temperature (T_ty_) and skin temperatures at four sites (chest, forearm, front thigh, and front shin) were continuously monitored with thermistors (Gram, LT-8, Tokyo, Japan) at 30-s intervals throughout the experimental period. Mean skin temperature ($\bar{\mathrm{T}}_{\mathrm{sk}}$) was calculated using Ramanathan’s equation [17]. Local sweat rates at four sites (chest, forearm, front thigh, and front shin) were monitored with local sweat rate monitors (OKS-04HM; SKINOS Co., Nagano, Japan) at the end of each exposure to heat. The mean sweat rate ($\bar{\mathrm{S}}_{\mathrm{w}}$, mg/cm^2^/min) was also calculated using Ramanathan’s equation assuming that Ramanathan's formula can be applied to estimate the mean value.

HR and the R-to-R interval were continuously monitored with a sampling rate of 500 Hz with an ECG (DAQ terminal; Intercross Co., Tokyo, Japan) during the experimental period. HR is generally recognized to increase when the magnitude of stress becomes greater. HRV power spectral analysis was performed to examine frequency components of minimum 60 s length, and the high-frequency components (HF, 0.15–0.45 Hz) and the low-frequency components (LF, 0.04–0.15 Hz) were specifically analyzed due to their association with parasympathetic nervous system activity. Fluctuations of HF are mediated solely by the parasympathetic nervous system whereas LF fluctuations are mediated by both the parasympathetic and sympathetic nervous systems [1]. We focused on the change in the LF:HF ratio for evaluating heat stress during exposure to mild heat. Based on regression analysis of the LF:HF ratio, heat stress may be estimated to become greater from a higher positive coefficient of X in a linear equation.

### Statistical analysis

The outcome variables at the end of each exposure to mild heat and the cooling period were analyzed by 2-way ANOVA with a repeated-measures design. For TSRs, thermal comfort responses (TCRs), T_ty_, and $\bar{\mathrm{T}}_{\mathrm{sk}}$ factors included condition (1, 2 or 3) and repetition (1st—5th). For significant main effects or interaction comparisons of physiological and psychological variables were made using one-tailed paired t-tests. *p* < 0.05 was considered significant after Bonferroni correction. To confirm a changing trend of thermal sensation responses during the cooling period and change in the HR throughout the experimental period, linear regression analysis was conducted. Criteria for strong correlation was set at R^2^ value of 0.36.

## Results

The experiments were performed from the 10th of August to the 10th of September 2018. According to the Japan meteorological agency (https://www.jma.go.jp/jma/indexe.html), the mean daily temperature and relative humidity in Nagoya city were 27.9 °C and 67.2% RH.

### Change in tympanic temperature, mean skin temperature, thermal sensation and comfort responses

**Fig. 2:**
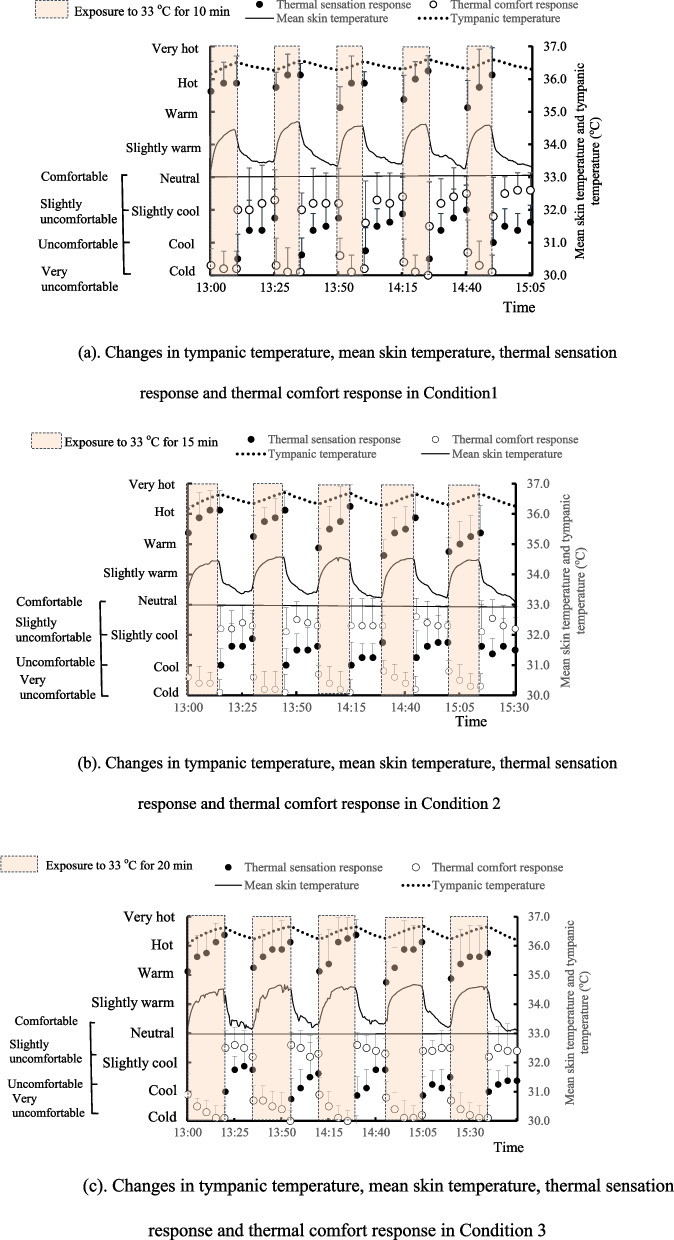
**a** Changes in tympanic temperature, mean skin temperature, thermal sensation response and thermal comfort response in condition 1. **b** Changes in tympanic temperature, mean skin temperature, thermal sensation response and thermal comfort response in condition 2. **c** Changes in tympanic temperature, mean skin temperature, thermal sensation response and thermal comfort response in condition 3. In all conditions, thermal sensation responses (TSRs) changed from “warm” to “hot” or “very hot” with the increase in mean skin temperature ($\bar{\mathrm{T}}_{\mathrm{sk}}$) during each exposure to mild heat. However, TSR changed from “cold” or “cool” to “slightly cool” and thermal comfort response changed from nearly “comfortable” to “slightly uncomfortable” while $\bar{\mathrm{T}}_{\mathrm{sk}}$ kept decreasing to 33 °C during the cooling period

The changes in T_ty_, $\bar{\mathrm{T}}_{\mathrm{sk}}$, TSR, and TCR under three conditions are indicated in Fig. 2 a, 2(b), and 2(c). Both $\bar{\mathrm{T}}_{\mathrm{sk}}$ and T_ty_ increased during each exposure to heat and decreased during each cooling period. There was no trend for the main effect of condition for $\bar{\mathrm{T}}_{\mathrm{sk}}$ and T_ty_ at the end of exposure to heat and the cooling period and significant repetition by condition interaction in all conditions. In all conditions, TSR changed from “warm” to “hot” with an increase in $\bar{\mathrm{T}}_{\mathrm{sk}}$ during each exposure to heat, whereas it changed from “cold” to “cool” with a decrease in $\bar{\mathrm{T}}_{\mathrm{sk}}$ during each cooling period. Thus, overshooting responses of thermal sensation after repeated exposure to heat were clearly observed in all conditions.

There was a trend for a main effect of condition (F = 4.25, *p* = 0.0168) for TSR at the beginning of the cooling period but no significant repetition by condition interaction. Post-hoc tests showed that TSR in condition 1 was significantly higher than condition 2 (*p* = 0.0132). TCR changed to “very uncomfortable” during each exposure to heat, whereas TCR changed to “slightly uncomfortable” during each cooling period. There was a trend for a main effect of condition for TCR at the beginning of the cooling period (F = 7.46, *p* < 0.001) and of exposure to mild heat (F = 4.26, *p* < 0.0166) but no significant repetition by condition interaction. Post-hoc tests showed that TCR in condition 1 was significantly lower than that in condition 2 (*p* = 0.0276) and condition 3 (*p* < 0.001).

### Relationship between the thermal sensation response and mean skin temperature

**Fig. 3:**
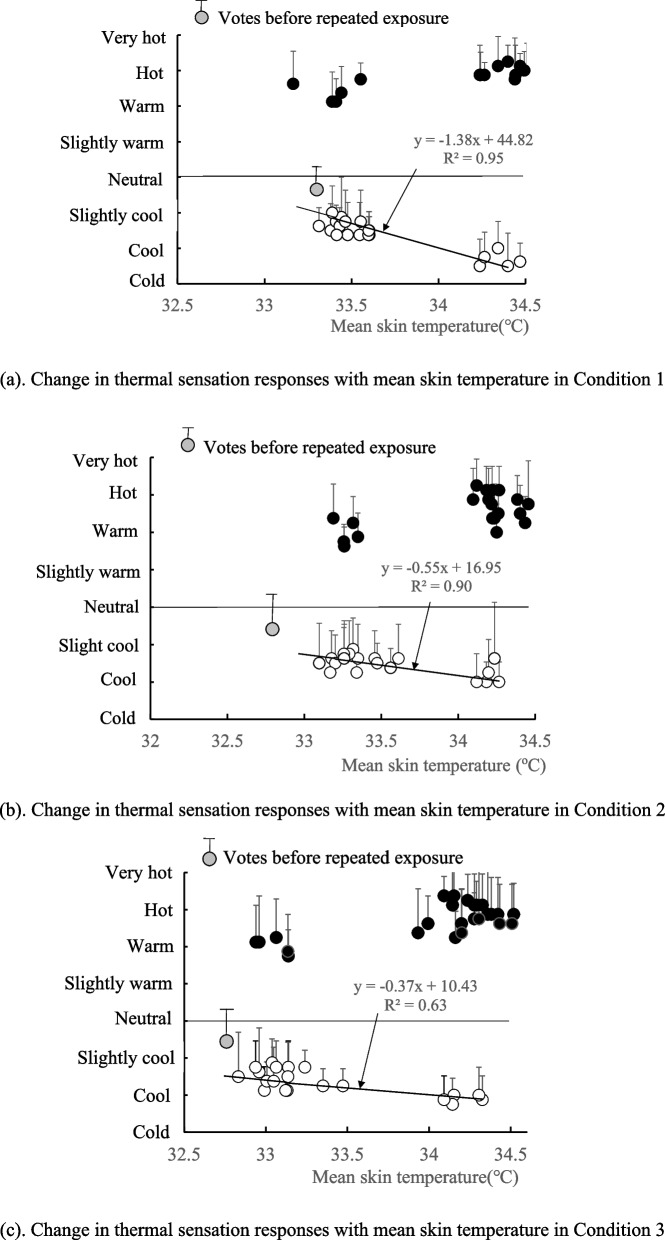
**a** Change in thermal sensation responses with mean skin temperature in condition 1. **b** Change in thermal sensation responses with mean skin temperature in condition 2. **c** Change in thermal sensation responses with mean skin temperature in condition 3. The figures show the relationship between thermal sensation responses (TSRs) and mean skin temperature ($\bar{\mathrm{T}}_{\mathrm{sk}}$) during the cooling periods. As indicated in Fig. 2a–c, the TSR changed from “cold” to “cool” or “slightly cool” with a decrease in $\bar{\mathrm{T}}_{\mathrm{sk}}$ during the cooling period in all conditions. Compared with TSR when the subjects stayed in the control room before repeated exposure, differences in TSR after exposure to mild heat can be clearly observed

The relationship between TSR and $\bar{\mathrm{T}}_{\mathrm{sk}}$ is indicated in Fig. 3 a–c. In all conditions, it was observed that the subjects voted “cold” at the beginning of the cooling period and voted “cool” at the end of the cooling period although $\bar{\mathrm{T}}_{\mathrm{sk}}$ continuously decreased. This relation of TSR to $\bar{\mathrm{T}}_{\mathrm{sk}}$ was opposite to the generally recognized relation (for example, [6]). The regression analysis on the change in TSR during the cooling period showed that the subjects were expected to vote “neutral” when $\bar{\mathrm{T}}_{\mathrm{sk}}$ decreased to 32.5 °C in condition 1, 30.7 °C in condition 2, and 28.6 °C in condition 3. The results indicated that the $\bar{\mathrm{T}}_{\mathrm{sk}}$ corresponding to “thermally neutral” after repeated exposure to mild heat may be strongly associated with the exposure time.

### Change in mean sweat rates during repeated exposure to heat

**Fig. 4:**
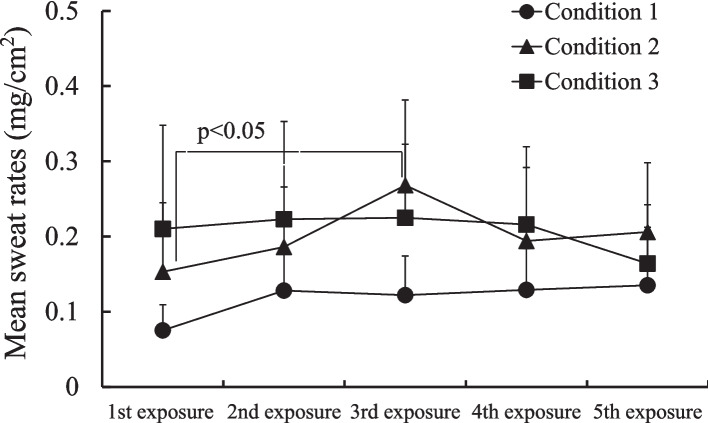
Change in mean sweat rates during exposure to mild heat. Mean sweat rates ($\bar{\mathrm{S}}_{\mathrm{w}}$) in condition 2 and condition 3 were significantly (*p* < 0.05) higher than those in condition 1

The change in mean sweat rates ($\bar{\mathrm{S}}_{\mathrm{w}}$) during exposure to heat is indicated in Fig. 4. Although there was no significant change in $\bar{\mathrm{S}}_{\mathrm{w}}$ due to repetition, $\bar{\mathrm{S}}_{\mathrm{w}}$ in condition 2 and condition 3 was significantly (*p* < 0.05) higher than in condition 1. Thus, $\bar{\mathrm{S}}_{\mathrm{w}}$ may be dependent on exposure time.

## Change in HR and LF:HF ratio during repeated exposure to mild heat

**Fig. 5:**
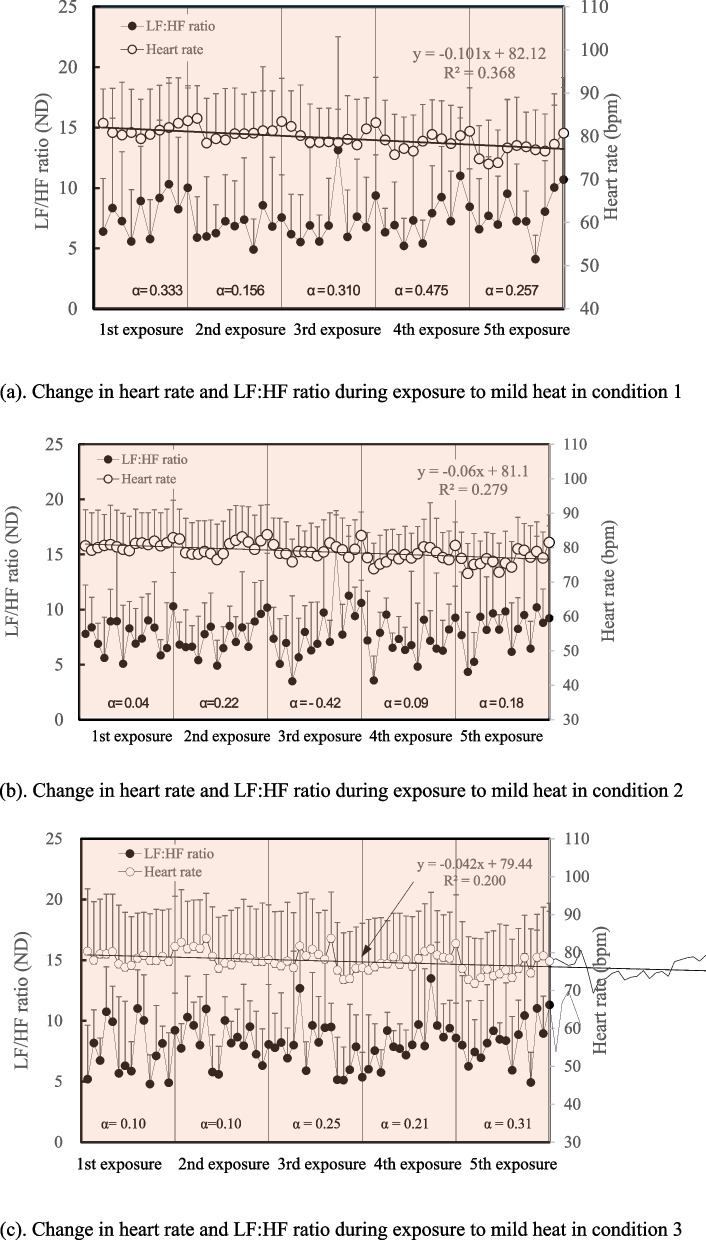
**a** Change in heart rate and LF:HF ratio during exposure to mild heat in condition 1. **b** Change in heart rate and LF:HF ratio during exposure to mild heat in condition 2. **c** Change in heart rate and LF:HF ratio during exposure to mild heat in condition 3. The regression analysis showed that the heart rate (HR) during repeated exposure to mild heat showed continuous decrease in all conditions although R^2^ values in condition 2 and condition 3 did not exceed the criteria for strong correlation. The decrease in HR appeared to be greater when exposure time was shorter. The LF:HF ratio increased during each exposure in all conditions since coefficients of X (α) in a linear equation were all positive. However, the α value continuously increased to the 4th exposure in condition 1 and then decreased to the 5th exposure. This may imply the effect of short-term heat acclimation

Change in HR and LF:HF ratio during repeated exposure to mild heat is indicated in Fig. 5 a–c. To estimate the trend of change in HR and LF:HF ratio, regression analysis was conducted. HR during repeated exposure to mild heat showed continuous decrease in all conditions. Although the correlation coefficients were low, the decreasing rate in HR appeared to be greater when exposure time was shorter. The LF:HF ratio increased during each exposure in all conditions since coefficients of X (α) in a linear equation were all positive. Although the α value continuously increased to the 4th exposure in condition 1 and to the 3rd exposure in condition 2, it then decreased to the 5th exposure.

## Discussion

Since Chen et al. [3] reported that overshooting responses were highly probable during the step change from 33 °C to 26 °C, the step change from 33 °C to 26 °C was adopted in the present study. As indicated in Fig. 2a–c, the subjects voted “cool” or “slightly cool” although $\bar{\mathrm{T}}_{\mathrm{sk}}$ remained higher than $\bar{\mathrm{T}}_{\mathrm{sk}}$, which considered to vote “warm” or “slightly warm”. Thus, overshooting responses in thermal sensation were observed repeatedly during redone exposure to mild heat. Since statistical analysis indicated that overshooting responses in condition 1 were significantly higher (*p* = 0.0132) than those in condition 2, overshooting responses after repeated exposure to mild heat may be related to the exposure time. In other words, the longer exposure may induce a larger difference in TSR before and after the step change in air temperature.

Regarding overshooting responses, other studies (Nagano et al., [10, 13, 14]) did not confirm the overshooting response of thermal sensation during a single step change in air temperature. Possible reasons may be sex differences [9, 21], seasonal differences [22] and effect of clothing. In the present study, the subjects wore a short-sleeved shirt and knee-length trousers. Since clothing absorbs sweat during heating, the heat loss pathway during cooling may become complicated compared with that of a nude subject. For example, the surface temperature of skin covered with clothing may not be promptly lowered at the beginning of cooling as compared with the surface temperature of unclothed skin. In relation to this issue, it was observed that the subjects voted “cold” at the beginning of the cooling period and voted “cool” or “slightly cool” at the end of the cooling period although $\bar{\mathrm{T}}_{\mathrm{sk}}$ continuously decreased. This relation of TSR to $\bar{\mathrm{T}}_{\mathrm{sk}}$ was opposite to the generally recognized relation but may correspond to the reality that people who stayed outdoor in summer preferred low air temperature soon after they moved into the air-conditioned space. As indicted in Fig. 3a–c, the generally recognized relation of $\bar{\mathrm{T}}_{\mathrm{sk}}$ to TSR may resume after a prolonged cooling period as predicted by Takada et al. [18] and the resuming time may be dependent on the exposure time.

Regarding short-term heat acclimation due to repeated exposure to heat, Fujii et al. [5] reported that acclimation appeared after seven days of exercise. In the present study, change in $\bar{\mathrm{T}}_{\mathrm{sk}}$ and T_ty_ indicated no short-term heat acclimation. $\bar{\mathrm{S}}_{\mathrm{w}}$ showed almost no change with repetition with the exception of condition 2, where $\bar{\mathrm{S}}_{\mathrm{w}}$ increased significantly (*p* < 0.05) from the 1 st to the 3rd exposure. Thus, $\bar{\mathrm{S}}_{\mathrm{w}}$ may be correlated with exposure time, but it indicated no short-term heat acclimation due probably to the amount of heat loss required during repeated exposure to heat was not sufficient to induce an increase in $\bar{\mathrm{S}}_{\mathrm{w}}$. Considering more severe heat stress, for example, a higher air temperature with a longer exposure time, which people experience almost every day in summer, short-term heat acclimation may be observed during repeated exposure to heat. However, Kakitsuba et al. [12] reported the results of a human experiment where young female subjects were exposed repeatedly to mild heat in the evaluation of their fatigue and demonstrated significant increase in $\bar{\mathrm{S}}_{\mathrm{w}}$ (*p* < 0.01) with repetition. So, it is necessary to consider sex differences in the sweating response during repeated exposure to heat.

Ohori et al. [16] and Vidyarini and Maeda [20] demonstrated that the HRV analysis may be a useful tool to estimate heat stress. Although heat stress may have been insufficient in the present study, change in HR suggested that the subjects may have been acclimated in condition 1 more effectively than in condition 2 and condition 3 because the decreasing rate in HR appeared to be greater when exposure time was shorter. Change in the LF:HF ratio also suggested that heat stress may be reduced during the latter half of repetition in condition 1 and condition 2 because the α value continuously increased to the 4th exposure in condition 1 and to the 3rd exposure in condition 2 and then decreased to the 5th exposure. Thus, short-term heat acclimation may be expected when exposure time to heat is coupled with the equal cooling time.

## Conclusion

During repeated exposure to mild heat coupled with the cooling period, it was observed that the subjects voted nearly “cold” when $\bar{\mathrm{T}}_{\mathrm{sk}}$ remained high at the beginning of the cooling period in all conditions. Thus, overshooting responses in thermal sensation were repeatedly observed. The subjects voted “slightly cool” at the end of cooling period while $\bar{\mathrm{T}}_{\mathrm{sk}}$ kept decreasing during the cooling period. The thermally neutral $\bar{\mathrm{T}}_{\mathrm{sk}}$ was then estimated to be 0.3 °C – 4.2 °C lower than $\bar{\mathrm{T}}_{\mathrm{sk}}$ observed prior to the first exposure. Thus, a residual effect on TSR during the cooling period was confirmed. Changes in mean sweat rate, TSR, and TCR showed significant differences between conditions but no indication of short-term heat acclimation. However, change in HR and ECG analysis implied the effect of short-term heat acclimation.

## Data Availability

No datasets were generated or analysed during the current study.
